# Clonal Origins of *Vibrio cholerae* O1 El Tor Strains, Papua New Guinea, 2009–2011

**DOI:** 10.3201/eid1711.110782

**Published:** 2011-11

**Authors:** Paul F. Horwood, Deirdre Collins, Marinjho H. Jonduo, Alexander Rosewell, Samir R. Dutta, Rosheila Dagina, Berry Ropa, Peter M. Siba, Andrew R. Greenhill

**Affiliations:** Papua New Guinea Institute of Medical Research, Goroka, Papua New Guinea (P.F. Horwood, D. Collins, M.H. Jonduo, P.M. Siba, A.R. Greenhill); University of Western Australia, Perth, Western Australia, Australia (D. Collins); World Health Organization, Port Moresby, Papua New Guinea (A. Rosewell); University of New South Wales, Sydney, New South Wales, Australia (A. Rosewell); Port Moresby General Hospital, Port Moresby (S.R. Dutta); National Department of Health, Port Moresby (R. Dagina, B. Ropa)

**Keywords:** bacteria, waterborne infections, enteric infections, Vibrio cholerae, cholera, VNTR, Papua New Guinea, MLST, clonal, outbreak, dispatch

## Abstract

We used multilocus sequence typing and variable number tandem repeat analysis to determine the clonal origins of *Vibrio cholerae* O1 El Tor strains from an outbreak of cholera that began in 2009 in Papua New Guinea. The epidemic is ongoing, and transmission risk is elevated within the Pacific region.

In July 2009, an outbreak of cholera began in the Morobe Province of Papua New Guinea (PNG) (*1*), and in the following months the disease spread throughout the coastal regions of the country. Although environmental and social conditions are conducive to the transmission and sustained presence of cholera, to our knowledge, this was the first outbreak of cholera in PNG. Sporadic outbreaks have occurred in the nearby Indonesian province of West Papua (formerly Irian Jaya) in the 1960s, 1990s, and, most recently, in 2008 (*2**,**3*). As such, conjecture has existed about whether this outbreak was the result of a new incursion of *Vibrio cholerae* or a reemergence of previously undetected strains endemic to PNG. We used multilocus sequence typing (MLST) and variable number tandem repeat (VNTR) analysis to investigate the diversity of the PNG *V. cholerae* strains and to elucidate the origin of this outbreak.

## The Study

The PNG cholera outbreak was first reported in Lambutina and Nambariwa villages in Morobe Province on the northeast coast of mainland PNG in July 2009 (*1*). The outbreak spread within the province and then to Madang and East Sepik Provinces along the northwest coast. In January 2010, the epidemic reached the national capital, Port Moresby, resulting in a large sustained outbreak in National Capital District and surrounding Central Province. In the following months, the outbreak spread along the south coast to Gulf and Western Provinces. Recently, the cholera outbreak has spread to the Autonomous Region of Bougainville. Since July 2009, >15,500 cases of cholera have been reported in PNG, with 493 recorded deaths (Figure).

**Figure:**
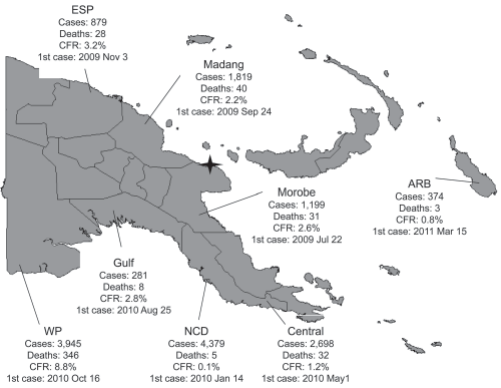
Cholera outbreak, Papua New Guinea, 2009–2011. Total cases: 15,582. Total deaths: 493. Overall case-fatality rate (CFR): 3.2%. Star denotes original outbreak sites of Lambutina and Nambariwa villages. ESP, East Sepik Province; ARB, Autonomous Region of Bougainville; WP, Western Province; NCD, National Capital District.

Clinical isolates from Morobe Province (n = 2), Madang Province (n = 2), Eastern Highlands Province (n = 2), East Sepik Province (n = 2), and National Capital District (n = 4) were isolated and identified as *V. cholerae* O1 El Tor Ogawa by using standard bacterial culture methods. The isolates were confirmed as *V. cholerae* serogroup O1 and carriers of the *ctxA* gene by multiplex PCR (*4*). All isolates were identified as altered El Tor through PCR detection of the classical *rstR* gene (*5*).

Nine loci were targeted for MLST analysis: *dnaE*, *lap*, *recA*, *pgm*, *gyrB*, *cat*, *chi*, *rstA*, and *gmd*, as described (*6*). The PCR products were visualized on an agarose gel, and direct sequencing was performed in both directions by using the MLST primers (Macrogen, Seoul, Korea). Contiguous nucleotide sequences were assembled by using Sequencher software (http://www.genecodes.com), and all nucleotide positions were confirmed by >2 independent sequencing reactions in each direction. The PNG MLST sequences were compared with previously reported sequences by using the same 9 loci outlined in previous studies (*5**–**7*). All PNG isolates displayed 100% nt identity across the 9 MLST loci and were identical to the Bangladesh strain MJ-1236 (*8*).

Five loci were analyzed for tandem repeats by using VNTR-specific primers, as described (*9*). The targeted regions were VC0147, VC0436–7, VC1650, VCA0171, and VCA0283. Contiguous nucleotide sequences were prepared as described above. Sequence types were designated by the actual number of repeats at the target loci as described in recent studies (*10**,**11*) and compared with sequence-derived VNTR data from the international literature and databases. Three sequence types were detected that were all within clonal complex 10,6,8,X,X (Table). The isolates from PNG were most closely related to strains from Vietnam (1995–2004) in the MLVA group III reported by Choi et al. (*12*). As reported, loci on the small chromosome (VCA0171 and VCA0283) were more variable than the loci on the large chromosome (*9**,**11**,**12*).

**Table:** Variable number tandem repeat profiles of Papua New Guinea *Vibrio cholerae* isolates and comparison with related international strains*

Isolate	Source and year	VC0147	VC0436–7	VC1650	VCA0171	VCA0283	Reference
M1	Madang, PNG, 2010	10	6	8	8	12	This study
M2	Madang, PNG, 2010	10	6	8	9	12	This study
G1	EHP, PNG, 2010	10	6	8	8	12	This study
G2	EHP, PNG, 2010	10	6	8	8	12	This study
L1	Morobe, PNG, 2010	10	6	8	8	11	This study
L2	Morobe, PNG, 2010	10	6	8	8	11	This study
W1	ESP, PNG, 2010	10	6	8	8	12	This study
W2	ESP, PNG, 2010	10	6	8	8	12	This study
P1	NCD, PNG, 2011	10	6	8	8	11	This study
P3	NCD, PNG, 2011	10	6	8	8	12	This study
P4	NCD, PNG, 2011	10	6	8	8	12	This study
P5	NCD, PNG, 2011	10	6	8	8	11	This study
07.95/Vc.P	Vietnam, 1995	10	6	8	16	26	(*12*)
272.03/Vc.P	Vietnam, 2003	10	6	8	17	28	(*12*)
43.04/Vc.P	Vietnam, 2004	10	6	8	16	29	(*12*)
MJ-1236	Bangladesh, 1994	8	7	8	12	19	(*12*)

*Variable number tandem repeat profiles indicate the actual number of repeats. PNG, Papua New Guinea; EHP, Eastern Highlands Province; ESP, East Sepik Province; NCD, National Capital District.

## Conclusions

The homogeneity of outbreak strains in PNG and the relatedness to strains from Vietnam by VNTR analysis is indicative of a recent incursion from the Southeast Asia region. It is unlikely that this outbreak is because of a previously undetected autochthonous endemic strain, given the close relationship with the isolates from Vietnam.

MLST and VNTR have been used by numerous research groups to analyze the diversity of outbreak strains and to investigate the origin of epidemics (*6**,**9**–**11*). MLST analysis of the *V. cholerae* strains and comparison with previously published MLST data suggested that the most closely related isolate was from Bangladesh (*8*). All isolates from PNG were identical to the strain MJ-1236 across the 9 housekeeping genes examined by sequence analysis. In contrast, the VNTR sequence analysis suggested that the PNG outbreak strains were most closely related to strains isolated from Vietnam in 1995, 2002, 2003, and 2004 (*12*). MLST data were not available for the Vietnam strains in the international literature or databases; therefore, a direct comparison cannot be made between the PNG MLST and VNTR results.

This outbreak highlights the continued challenge that cholera presents to authorities worldwide: the disease can spread rapidly and the causative organism persists in the environment (*13*), which makes prevention and control of the disease complex. In PNG the large estuarine waterways (e.g., Sepik and Fly Rivers) and the settlement areas (which are often in estuarine areas with limited water and sanitary infrastructure and are more densely populated than rural and urban areas) present potential reservoirs for *V. cholerae*. The prevalence of enteric diseases remains high in PNG where access to safe drinking water is limited, particularly in rural areas where an estimated 87% of the population lives (*14*). These factors may aid the persistence of *V. cholerae* and result in a transition to endemicity of cholera in PNG.

During this outbreak, a relatively high national case-fatality ratio (CFR) of 3.2% was recorded. The provincial CFRs varied widely from 0.1% in the National Capital District, where oral rehydration salts points and treatment centers provided timely accessible treatment, to 8.8% in Western Province, where health system access and preparedness were weak. Strong leadership and coordination contributed to effective response but were limited where CFRs were high.

Despite road networks linking affected coastal areas to the mountainous interior, where most of the country’s population resides, imported cases have not resulted in ongoing transmission. This lack of transmission may be related to a less favorable habitat for environmental persistence of the organism and ongoing transmission. A similar situation was reported from the current outbreak of cholera in Haiti, where location on a coastal plain was a notable risk factor for cholera cases (*15*). Nonetheless, cholera remains a high risk for both affected and unaffected provinces in PNG. Moreover, the frequent international migration between PNG and neighboring communities with no prior cholera exposure and with vulnerable sanitary conditions heightens the risk for international spread.

Although the MLST and VNTR results concur that the PNG strains are closely related, our data suggest that VNTR has greater discriminatory power when used for investigations into the clonality and relatedness of *V. cholerae* strains. Other studies have highlighted the value of VNTR for strain typing of *V. cholerae* (*9**–**12*), but reports that directly compare VNTR and MLST are lacking. However, analysis by either VNTR or MLST is hampered by the paucity of data available to compare outbreak strains from around the world. An online *V. cholerae* VNTR database would enable more accurate tracking of the evolution of outbreaks and provide evidence for the mode of spread of *V. cholerae* strains between countries and geographic areas. A more comprehensive analysis of *V. cholerae* strains from around the world is also required to gain a better understanding of the global and regional spread of these strains.
